# Increased Susceptibility of Cattle to Intranasal RVFV Infection

**DOI:** 10.3389/fvets.2020.00137

**Published:** 2020-04-29

**Authors:** Andrea L. Kroeker, Valerie Smid, Carissa Embury-Hyatt, Brad Collignon, Mathieu Pinette, Shawn Babiuk, Bradley Pickering

**Affiliations:** ^1^Canadian Food Inspection Agency, National Centre for Foreign Animal Disease, Winnipeg, MB, Canada; ^2^Department of Immunology, University of Manitoba, Winnipeg, MB, Canada; ^3^Department of Medical Microbiology, University of Manitoba, Winnipeg, MB, Canada; ^4^College of Veterinary Medicine, Iowa State University, Ames, IA, United States

**Keywords:** virus, Rift Valley Fever, cattle, animal model, Phenuiviridae

## Abstract

Rift Valley Fever virus (RVFV) is a zoonotic mosquito-borne virus that belongs to the Phenuiviridae family. Infections in animal herds cause abortion storms, high mortality rates in neonates, and mild to severe symptoms. Infected animals can also transmit the virus to people, particularly people who live or work in close contact with livestock. There is currently an ongoing effort to produce safe and efficacious veterinary vaccines against RVFV in livestock to protect against both primary infection in animals and zoonotic infections in people. To test the efficacy of these vaccines it is essential to have a reliable challenge model in relevant target species, including ruminants. In this study we evaluated three routes of inoculation (intranasal, intradermal and a combination of routes) in Holstein cattle using an infectious dose of 10^7^ pfu/ml and a virus strain from the 2006–2007 outbreak in Kenya and Sudan. Our results demonstrated that all routes of inoculation were effective at producing viremia in all animals; however, the intranasal route induced the highest levels and longest duration of viremia, the most noticeable clinical signs, and the most widespread infection of tissues. We therefore recommend using the intranasal inoculation for future vaccine and challenge studies.

## Introduction

Rift Valley Fever virus (RVFV) is a single-stranded RNA virus that belongs to the Phenuiviridae family. It was first described in Eastern Africa in the early 1900s (1) and initially drew attention during animal outbreaks that resulted in high rates of abortion. Since it was first detected, RVFV has spread to new regions and continues to circulate widely throughout much of Africa (2, 3). Serosurveys have demonstrated the presence of antibodies against RVFV in a variety of animal species including domestic ruminants such as sheep, goats, cattle, alpacas and camels in addition to a variety of wildlife such as the African buffalo (2, 4–8). Interestingly, these serosurveys have shown that RVFV circulates not only during outbreaks but also during inter-epidemic periods including areas where outbreaks have never occurred (9, 10). Although the number of seropositive animals varies widely based on timing and region, where seropositivity ranges from 0 to 100% in sheep and cattle, to 0–50% in goats and 0–30% in camels and humans (2, 3), these studies clearly highlight the important role that animals play in the evolution and spread of RVFV.

Despite its widespread presence in Africa, RVFV outbreaks only occur sporadically and do not necessarily occur in every area with seropositive animals. Outbreaks typically occur during periods of increased rain which are associated with an increase in mosquito populations (11). In ruminants, outbreaks are characterized by abortion storms and high rates of mortality, especially in neonates. Although mortality rates can vary significantly between different outbreaks, during the South African outbreak in 2010–2011, adult cattle, sheep and goats had an estimated 50–62% mortality rate while camels, buffaloes and other wildlife species experienced 100% mortality (12). Other studies have also reported high rates of abortions such as 70% in sheep and goats during out outbreak in Mauritania in 2003 (13).

RVFV outbreaks also pose significant risks to human populations. Susceptible animals such as ruminants amplify the virus to titers that are high enough to transmit to humans and are one of the primary reservoirs for human infections. The major risk factors associated with RVFV infections in humans are related to close proximity with livestock, including animal husbandry, animal slaughtering and exposure to raw milk (14–18). In addition to health risks, the loss of fetuses and newborn livestock to RVFV infections can have a severe socio-economic impact on farmers (19). Together this data suggests that vaccinating livestock against RVFV may be highly beneficial not only in protecting livestock but also to the people who are in direct contact with them (20, 21). Since the risk of human infections increases as the seropositivity increases in animal populations (22), surveillance systems in countries where RVFV circulates are extremely important. Although RVFV surveillance data for many African or other at-risk countries is currently sparse, the development of international surveillance networks (23–26) will make it much easier to monitor and share data regarding the presence of RVFV. Surveillance data will also be useful for informing vaccination programs about areas requiring preferential targeting. These regions should also be studied to identify potential barriers against uptake of the vaccine; for example, limited health education and cost of the vaccine have prevented vaccination of livestock in the past (27).

There are currently several RVFV veterinary vaccine options available to African farmers such as formalin-inactivated vaccines and the Smithburn vaccine. However, the formalin-inactivated vaccine is inadequate at preventing viremia (28) and safety issues have been identified with the live attenuated Smithburn vaccine (29), which have stimulated the development of several new RVFV vaccines (30). Some of the new RVFV vaccines have already undergone safety and immunogenicity testing in sheep such as a four-segmented RVFV vaccine (31), a Gn subunit vaccine (32), a DNA vaccine containing either GP and NP genes (33), a non-spreading (NSR) RVFV vaccine (34) and an equine herpesvirus type 1 vector (35); others have been tested for safety and immunogenicity in other natural host species including a Gn-based vaccine with a paramyxovirus vector in sheep and calves (36, 37), a Gn-based vaccine with a modified vaccinia Ankara vector in sheep and baboons (38, 39), a Gn-based vaccine with a Chimpanzee adenovirus vector in sheep, calves and camels (40), a Gn-based vaccine with capripox vector (33, 41, 42), MP12 in sheep, goats and cattle (43), and Clone 13 in sheep, goats, calves and camels (44–50). In terms of efficacy, many novel RVFV vaccines have proven efficacious in mouse models; however, as of yet, only a few efficacy challenges have been performed in ruminants: a Gn subunit vaccine (51), R566 (52) and non-spreading vaccine were 100% efficacious in sheep (52), and Clone 13 was 100% efficacious in sheep (45) and cattle (50).

Recently, in partnership with Kansas State University, we sought to develop optimal RVFV infections in ruminants to provide tools for evaluation of vaccines. These include sheep (53, 54), goats (54–56), and cattle (57) which were tested using a variety of factors such as different virus doses, viral strains, routes of inoculation, and animal breeds. While sheep and goats had consistent viremia, cattle proved to be more resistant to infection as only 2 out of 5 animals developed robust viremia (57). Therefore, the aim of this study was to develop a robust RVFV infection with an increased proportion of cattle with viremia.

The experimental design of this study was based on the cattle model developed at Kansas State University (57) as well as previous sheep and goat model development at the NCFAD (54–56), but with several adaptations. Holstein calves were used instead of the Angus breed, although the age range of the animals was similar and at an appropriate age for vaccination (4–6 months). A unique virus isolate from the Kenya/Sudan 2006–2007 outbreak, previously characterized in goats [RVFV-UAP (55), at a slightly higher inoculation titer (10^7^ pfu instead of 10^6^ pfu). The RVFV-UAP virus isolate was chosen for the goat study because the Wilson group had had good success with the Ken06-128b isolate in terms of inducing viremia and systemic spread to the tissues, including liver lesions and detection of virus in the brain (57). However, due to the complexities in shipping live viruses between countries, the RVFV-UAP isolate was evaluated instead. Since the RVFV-UAP isolate and the 10^7^ pfu dose proved to be robust in goats (55), it was used in the current study. The inoculation titer of 10^7^ pfu was chosen based on previous sheep and goat model data from NCFAD (54, 55). Different routes of inoculation were evaluated. Whereas, Wilson et al. had used subcutaneous inoculation which is widely utilized in the literature, it was previously demonstrated that the intranasal route could induce higher levels of viremia in goats than the subcutaneous route (55) and therefore it was hypothesized that the intranasal route may work well in cattle as well. Although most of the literature utilizes subcutaneous injections, it is possible that a different subset or a greater number of dendritic cells could be infected by intradermal injection. For example, dendritic cells have been shown to infiltrate the dermis upon infection and play a role in presenting antigens from skin vaccinations and infections (58, 59). In addition, a combination of routes was used (10^7^ pfu subcutaneous, 10^7^ pfu intradermal and 10^7^ pfu intranasal) in the anticipation that cattle could be fairly resistant to infection and may require more than just a single injection. It was previously demonstrated that intravenous injection was not any better than a subcutaneous injection at inducing viremia (54), and similarly, inoculating twice (once on day 1 and a second inoculation on day 2) did not increase viremia titers over and above a single inoculation (54). By modifying the parameters from the previous RVFV challenge models, we sought to increase the robustness of the cattle challenge model to more effectively test RVFV vaccines and to minimize the number of animals needed to produce statistically relevant vaccine efficacy data.

## Results

### Clinical Signs, Temperature, Clinical Chemistry, and Viremia

**Figure 1:**
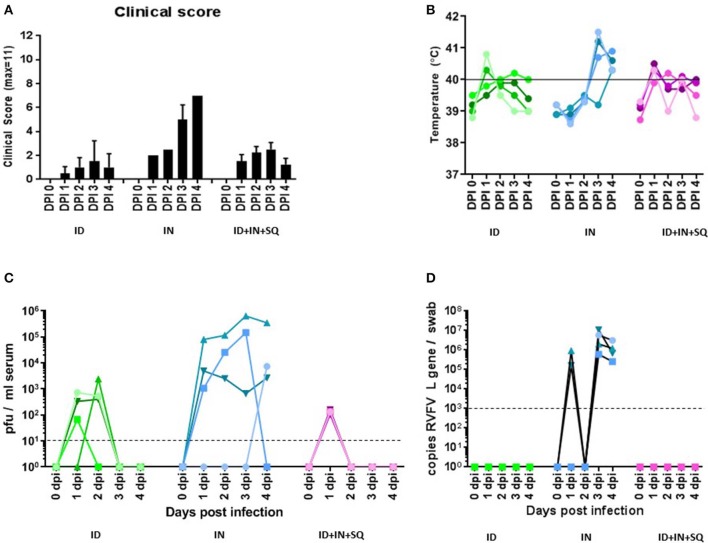
Clinical signs, viremia, and shedding. **(A)** All animals were assessed daily for signs of disease, rectal temperature, eating and drinking habits, disposition and stool consistency and given a clinical score. The average clinical score per group of animals (*n* = 4) is shown. **(B)** Rectal temperatures for each animal on a daily basis; each value represents an individual animal. **(C)** Infectious virus was measured in the blood by plaque assay on a daily basis; each value represents an individual animal. The horizontal dashed line indicates the detection limit of the plaque assay. **(D)** Viral RNA was measured in nasal swabs by RT-PCR on a daily basis; each value represents an individual animal. The horizontal dashed line indicates the diagnostic detection limit of the RT-PCR assay.

Throughout the experiment, animals were evaluated for clinical signs of disease on a daily basis. The clinical score was a sum of the animals' general appearance, rectal temperature, alertness, eating and drinking habits, and stool consistency. The endpoint was defined as reaching a clinical score of 11, not eating or drinking for more than 24 h or for any other unforeseen cause identified by the institutional veterinarian. For the intradermal and combination groups, the signs of disease were very mild with clinical scores of 1.3 to 2.5 after inoculation (Figure 1A); with the exception of a mild fever around 39.5–40°C (Figure 1B), the animals were generally asymptomatic. In contrast, the intranasal inoculation produced mild but noticeable clinical signs with a clinical score of 6 and 7 on days 3 and 4 (Figure 1A), which was accompanied by a more pronounced fever between 40–41°C (Figure 1B). A summary of the individual scoring data can be found in Supplementary Table 1.

Viremia was measured daily using plaque assays. All animals in all groups became viremic, although the duration and level of viremia varied. In the intradermal group, infectious virus was detected on days 1 and 2 with peak levels of 10^3^ pfu/ml serum (Figure 1C). The combination group generated 10^2^ pfu/ml of infectious virus only on day 1 (Figure 1C). The intranasal group developed the highest levels of virus, showing viremia on all 4 days with infectious virus ranging from 7 ×10^2^ to 6 ×10^5^ pfu/ml serum (Figure 1C) with peak levels on day 3 or 4.

In addition, a clinical biochemistry panel was performed on the serum to evaluate the impact of RVFV infection on organ function. Mild increases were observed in ALB, TP, ALP, CA in all groups and additional increases in BUN were seen in the intranasal group. The average values for each group are listed in Tables 1–3, and the individual data can be viewed in Supplementary Table 2.

**Table 1:** Average serum clinical chemistry values for the intradermal inoculation group.

	**Normal range**	**DPI 0**	**DPI 1**	**DPI 2**	**DPI 3**	**DPI 4**
ALB	2.5–3.8	3.40	4.68	5.18	3.95	3.90
ALP	23–135	127.50	201.25	236.75	160.25	145.50
AST	66–211	70.25	108.50	129.00	75.75	67.75
CA	7.9–9.6	10.75	13.73	15.33	11.88	11.70
GGT	12–48	16.50	22.75	28.00	18.50	20.00
TP	6.6–9.3	7.60	9.20	11.13	7.63	7.50
GLOB	4.4–5.5	3.50	4.50	5.45	3.68	3.60
BUN	6–20	14.25	12.50	15.25	10.75	9.25
CK	83–688	320.00	437.25	482.50	363.00	252.50
PHOS	4.1–9.2	7.00	9.00	10.40	7.48	7.60
MG	1.7–2.9	2.38	2.73	3.25	2.30	2.28

**Table 2:** Average serum clinical chemistry values for the intranasal inoculation group.

	**Normal range**	**DPI 0**	**DPI 1**	**DPI 2**	**DPI 3**	**DPI 4**
ALB	2.5–3.8	3.68	4.80	3.98	3.78	4.38
ALP	23–135	136.00	181.00	138.00	137.25	183.00
AST	66–211	63.50	99.25	78.50	142.25	189.25
CA	7.9–9.6	11.28	13.97	12.00	11.00	12.55
GGT	12–48	89.00	100.00	75.75	68.25	79.75
TP	6.6–9.3	7.33	10.40	7.75	7.35	9.00
GLOB	4.4–5.5	3.65	5.63	3.78	3.55	4.60
BUN	6–20	16.50	22.75	16.75	18.75	24.75
CK	83–688	281.50	444.50	554.50	312.25	393.25
PHOS	4.1–9.2	6.73	8.23	7.30	5.70	8.33
MG	1.7–2.9	2.28	3.13	2.33	1.98	2.50

**Table 3:** Average serum clinical chemistry values for the combination inoculation group.

	**Normal range**	**DPI 0**	**DPI 1**	**DPI 2**	**DPI 3**	**DPI 4**
ALB	2.5–3.8	3.68	4.49	5.28	4.63	4.37
ALP	23–135	136	143.38	234.92	174.08	159.25
AST	66–211	63.5	87.00	105.42	86.25	81.08
CA	7.9–9.6	10.01	12.15	11.30	7.21	12.80
GGT	12–48	16.5	20.94	22.17	18.08	17.83
TP	6.6–9.3	7.33	9.17	11.01	9.19	8.56
GLOB	4.4–5.5	3.65	4.67	5.71	4.54	4.19
BUN	6–20	16.5	15.88	23.17	18.75	18.83
CK	83–688	281.5	306.50	328.08	291.50	284.58
PHOS	4.1–9.2	6.73	9.93	10.84	9.02	9.28
MG	1.7–2.9	2.28	2.74	3.43	2.54	2.63

### Shedding and Mucosal Immunity

No infectious virus was isolated in the nasal and oral swabs at any time or in any group, and no viral RNA was detected in any of the oral swabs. However, viral RNA was observed in nasal swabs by qRT-PCR in the intranasal group with levels between 10^5^ and 10^7^ copies/swab at days 1, 3, and 4 post infection (Figure 1D).

**Figure 2:**
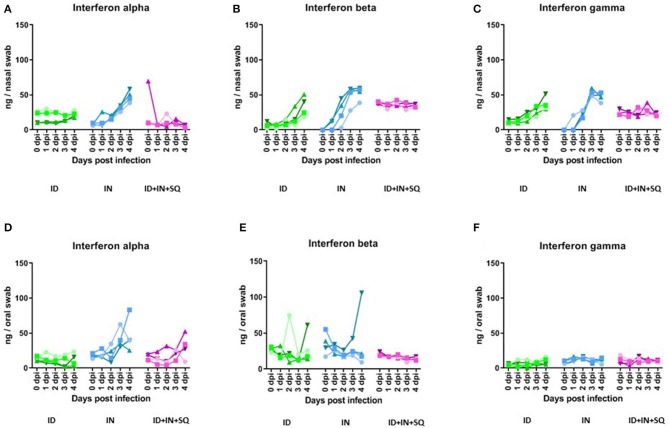
Interferons alpha, beta and gamma in nasal and oral swabs. IFN-α **(A,D)**, IFN-β **(B,E)**, and IFN-γ **(C,F)** were measured in nasal and oral swabs by ELISA; each value represents an individual animal. The horizontal dashed line indicates the diagnostic detection limit of the RT-PCR assay.

Due to the lack of infectious virus despite high levels of viral RNA in the nasal swabs, it was hypothesized that any virus in the nasal cavity had been inhibited directly, for example through the antiviral action of interferons on cells in the nasal cavity. Therefore, ELISAs were performed to monitor the levels of interferons alpha (IFN-α), beta (IFN-β) and gamma (IFN-γ) in the swabs. In the intranasal group, IFN-α, IFN-β, and IFN-γ were all detected in the nasal swabs starting at 1 or 2 dpi and peaked at 40–60 ng/swab at 3 or 4 dpi (Figures 2A–C); in comparison, the oral swabs from the intranasal group contained similar amounts of IFN-α but did not contain significant amounts of IFN-β or IFN-γ, except in one animal (Figures 2D–F). The intradermal inoculation also contained levels of IFN-β and IFN-γ in the nasal swabs, with peak levels at 20–50 ng/swab at 4 dpi, but not IFN-α (Figures 2A–C); in comparison, the oral swabs from two animals in the intradermal group had increased IFN- β after infection, while IFN-α and IFN-γ did not change from baseline (Figures 2D–F). We did not detect INFs in the nasal or oral swabs from the combined inoculation route group except for a low level of INF-α at 3–4 dpi (Figure 2E).

### Infection of Tissues and Pathology

The endpoint was determined by the parameters chosen to compare future vaccinated and non-vaccinated groups including: viremia, virus isolation from tissues, changes in blood chemistry, viral shedding in swabs, clinical signs and liver pathology. If present, all of these parameters should be detectable throughout the acute phase of infection (usually within the first week after infection); however, based on data from Wilson's cattle model, infectious virus is only present in the tissues at days 3 and 4 post infection (57); therefore 4 dpi was chosen as the endpoint. Inspection of the animals at necropsy did not indicate any gross pathology, except for animal #1835 in which we noted significant fibrosis in the liver. Tissues were collected fresh to evaluate viral loads using virus isolation or placed into formalin for sectioning to identify lesions if present.

**Figure 3:**
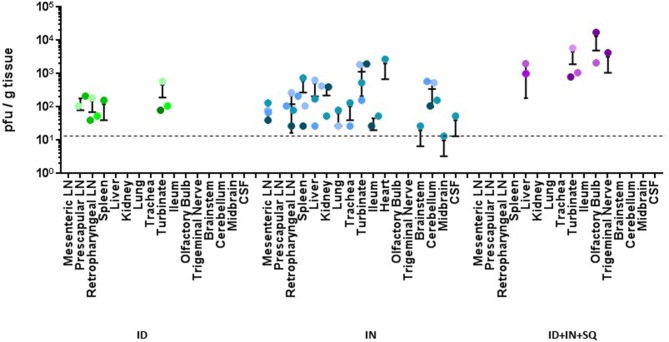
Virus load in tissues. Infectious virus was measured in tissues by plaque assay; each value represents an individual animal. All samples were run, but negative results were not included on the graph. The horizontal dashed line indicates the detection limit of the plaque assay.

The intradermal and combination groups presented the fewest number of tissues infected by the virus; the intradermal group harbored virus in the spleen, turbinates, prescapular lymph nodes, and retropharyngeal lymph node (Figure 3, ID group) and the combination group contained virus in the liver, turbinates, olfactory bulb and trigeminal nerve (Figure 3, ID-IN-SQ group). In contrast, the intranasal group had the greatest number of tissues infected by RVFV; infectious virus was isolated from mesenteric and retropharyngeal lymph nodes, spleen, liver, lung, trachea, turbinate, ileum, heart, brainstem, cerebellum, midbrain, and cerebral spinal fluid (Figure 3, IN group). Some of the tissues were consistently infected in all four animals within a group; however, some tissues were only infected in one or two animals within a group, in line with the variability between animals that is commonly seen in livestock.

**Figure 4:**
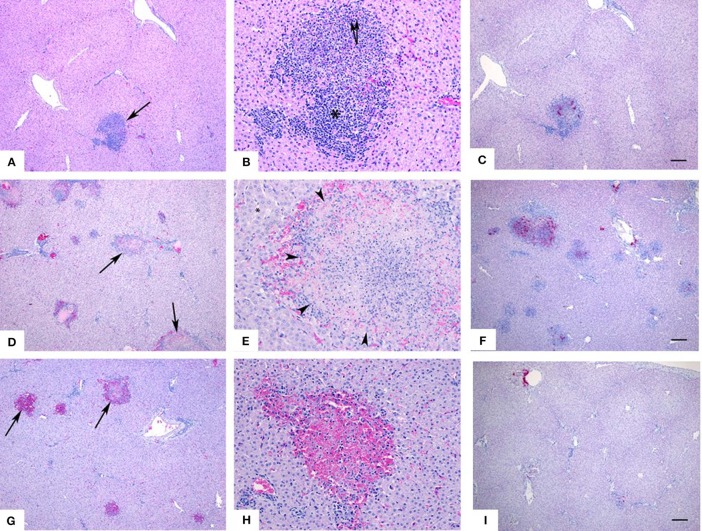
Liver histopathology and *in situ* hybridization. Livers from animals in the ID-IN-SQ combined inoculation group had small numbers of lesions (**A**, arrow) which on higher magnification **(B)** were characterized by areas of hepatocyte necrosis (arrows) and loss with replacement by a mixed inflammatory infiltrate (*). Most livers from intranasally inoculated animals had numerous lesions (**D**, arrows) which on higher magnification **(E)** were characterized by replacement of normal hepatocytes (*) with large areas of necrosis (delineated by arrows). Numerous lesions were also observed in several livers from the intradermally inoculated group (**G**, arrows). In this group there was significant hemorrhage associated with the areas of hepatocyte loss **(H)**. The presence of RVFV in the lesions was confirmed using *in situ* hybridization, in bright pink **(C,F,I)**.

Livers from all 3 groups of inoculated animals had lesions that were consistent with RVFV infection; however, they differed slightly in severity and stage of pathogenesis. Livers from animals in the ID-IN-SQ combined inoculation group had small numbers of lesions (Figure 4A, arrow, calf 1818) which on higher magnification (Figure 4B) were characterized by areas of hepatocyte necrosis (arrows) and loss with replacement by a mixed inflammatory infiltrate (^*^); the presence of RVFV in the lesions was confirmed using *in situ* hybridization (Figure 4C, calf 1818). Most livers from the intranasally inoculated animals had numerous lesions (Figure 4D, arrows, calf 1836) which on higher magnification (Figure 4E) were characterized by replacement of normal hepatocytes (^*^) with large areas of necrosis (delineated by arrows); the presence of RVFV lesions was confirmed using *in situ* hybridization (Figure 4F, calf 1836). Numerous lesions were also observed in several livers from the intradermally inoculated group (Figure 4G, arrows, calf 1912). In this group there was significant hemorrhage associated with the areas of hepatocyte loss (Figure 4H); the presence of RVFV in the lesions was confirmed using *in situ* hybridization (Figure 4I, calf 1912). In contrast, no lesions were found in the spleen of any animal.

## Methods

### Ethics Statement

All animal experiments were carried out in the enhanced biosafety level 3 (BSL3) facility at the National Centre for Foreign Animal Disease (NCFAD) in Winnipeg, Manitoba. All protocols for animal use were approved under the animal document use number C-17-002 at the Canadian Science Centre for Human and Animal Health (CSCHAH) in Winnipeg, Manitoba by the Animal Care Committee. Care was taken to minimize animal suffering and to follow the Canadian Council on Animal Care guidelines for animal manipulations.

### Cells

Mosquito C6/36 cells (ATCC, USA) were grown and infected in 1:1 EMEM and ESF-921 (Expression Systems, USA) supplemented with 10% fetal bovine serum (FBS, Hyclone) and 1% L-glutamine and maintained at 28°C without CO_2_. Mammalian Vero E6 (VE6) cells were grown and infected in DMEM (Gibco) supplemented with 10% FBS and maintained at 37°C with 95% relative humidity and 5% CO_2_.

### Virus Production and Titration

VE6 cells were infected with a virus isolate from the 2006–2007 Kenyan outbreak (RVFV-UAP; Genbank #MH175203, MH175204, MH175205) (55) at an MOI 0.1 and maintained in DMEM with 10% FBS. Thereafter, virus was alternatively propagated between VE6 and C636 cells twice. All passages were titrated on VE6 cells with a plaque assay to determine virus concentration. The calves were then infected using passage 6 C6/36-derived virus.

### RVFV Inoculation of Cattle

Twelve Holstein calves (3–4 months) were inoculated with RVFV-UAP that was grown in mosquito cell culture. Group 1 (*n* = 4) received intradermal (ID) inoculation; group 2 (*n* = 4) received intranasal (IN) inoculation; and group 3 (*n* = 4) received a combination (SQ/ID/IN) of all three routes. The subcutaneous injections consisted of 1 ×10^7^ pfu in 100 μl PBS in the left flank; the intradermal inoculations consisted of five injections of 2 ×10^6^ pfu in 100 μl PBS each in the left lumbosacral region; and the intranasal inoculations consisted of 1 ×10^7^ pfu in 1 ml PBS with half in each nostril.

### Sampling

All calves were carefully monitored for signs of illness and rectal temperature on a daily basis. We also collected serum on a daily basis and stored at −70°C. Nasal and oral swabs were collected on a daily basis, placed into 2 ml sterile PBS containing antibiotics and an antifungal and stored at −70°C.

### Clinical Chemistry

Serum biochemistry was evaluated daily with the VetScan VS2 blood analyzer (Abaxis, USA) and Large Animal Profile rotors (Abaxis, USA). All assays were run as per manufacturer's instructions and the bovine reference ranges were provided by Abaxis.

### Post-mortem Tissue Collection

At 4 days post infection we examined the calves for changes in gross pathology and collected fifteen tissues including liver, spleen, kidney, lung, ileum, retropharyngeal lymph node, prescapular lymph node, mesenteric lymph node, cerebral spinal fluid, brainstem, midbrain, cerebellum, olfactory bulb and trigeminal nerve. Separate pieces of each tissue were collected fresh and subsequently frozen at −80°C or placed in 10% formalin.

### Tissue Homogenization

We made 10% homogenates of each tissue by placing 5 g of tissue in a 7 ml PreCellys tube and adding 5 ml with PBS. This sample was then homogenized for 30 s at maximum speed using the Personal Homogenizer. A single homogenate was used for both downstream qRT-PCR and plaque assays without any freeze/thaw cycles.

### Virus Isolation From Oral and Nasal Swabs

Virus was isolated from oral and nasal swabs using two blind passages in 95% confluent monolayers of Vero E6 cells: 200 μl of each swab was adsorbed to cells in 24-well plates for 1 h at 37°C with gentle rocking, then overlaid with 1 ml serum-free DMEM, incubated for 7 days, and were visually checked for cytopathic effects. The entire contents from each well were then transferred to cells in T25 flasks, adsorbed to cell for 1 h at 37°C with gentle rocking, then overlaid with 4 ml serum-free DMEM, incubated for a further 7 days, and were visually checked for cytopathic effects.

### Virus Quantitation by qRT-PCR

RVFV RNA was extracted from serum using the TriPure Isolation Reagent (Roche) according to the manufacturer's instructions. Purified RNA was stored at −70°C. Viral RNA was detected using the TaqMan Fast Virus 1-Step RT-PCR master mix as per manufacturer's instructions and ran the samples on the ABI 7500 thermocycler with the following conditions: 5 min at 50°C, 2 min at 95°C and 40 cycles of 3 s at 95°C and 30 s at 60°C. Primers (Invitrogen) and probe (Biosearch) targeted nucleotides 2912 to 3001 for the RVFV L gene segment. All Ct values were plotted on a standard curve using a DNA plasmid containing the targeted RVFV L gene segment (GenScript) and quantified.

### Virus Quantitation by Plaque Assay

Serial dilutions of serum, nasal swabs, oral swabs and 10% tissues homogenates were used to infect confluent monolayers of VE6 cells in 48-well plates. Seventy five microliter of inoculum was added to the cells in triplicate for 1 h at 37°C with rocking. The inoculum was then removed and the cells were overlayed with 2 ml 1.75% carboxymethylcellulose (CMC). After 4 days the cells were formalin-fixed and stained with 0.5% crystal violet (Sigma) to visualize and count plaques.

### ELISA for Interferons

We used bovine interferon alpha (IFN-αA), beta and gamma ELISA kits (Kingfisher Biotech Inc., USA) to detect protein in nasal and oral swabs. 96-well MaxiSorp ELISA plates (Nunc) were coated with 2.5 ng/ml capture antibody diluted in DPBS and incubated at room temperature for 24 h. The plates were blocked with DPBS+4% bovine serum albumin at room temperature for 1 h. Oral and nasal samples were diluted 1:2 in DPBS before plating, standards were diluted in DPBS+4% bovine serum albumin and plates were incubated at room temperature for 1 h. Detection antibody was diluted in DPBS +4% bovine serum albumin at room temperature for 1 h, followed by 5 washes in TBS-Tween20 (0.05%). Plates were then incubated with Streptavidin-HRP at room temperature for 1 h, followed by 5 washes in TBS-Tween 20 (0.05%). TMB was added for colorimetric development, followed by 2N sulfuric acid as a stop solution. Plates were read on an Epoch (Biotek) plate reader at 450 nm.

### Tissue Sectioning and Staining

Five-micron paraffin-embedded formalin fixed tissue sections were cut, air-dried, and melted onto charged slides in a 60°C oven. The slides were then cleared and hydrated in xylene and 100% ethanol, and then air-dried. The sections were stained with hematoxylin and eosin (H&E) and imaged with a Zeiss microscope at 40X and 200X.

### *In situ* Hybridization

For the ISH technique, 5 um paraffin-embedded formalin fixed tissue sections were cut, air dried then melted on to the charged slides in a 60°C oven. Then the slides were cleared and hydrated in xylene and 100% ethanol then air dried. The sections were quenched for 10 min in aqueous H_2_O_2_, boiled in target retrieval solution for 15 min, rinsed in 100% ethanol and air dry again. Then a final pre-treatment of protease plus enzyme for 15 min at 40°C was applied. The probe (V-RVFV-ZH501-NP, from Advanced Cell Diagnostics) was applied and incubated at 40°C for 2 h. Then the Hybridization amplification steps (AMP 1-6) are applied to the slides for the recommended times and temps as per the manual for the RNAscope® 2.5HD Detection Reagent – Red kit (ACD). The signal is then visualized by the chromogen Fast Red. The sections were then counter stained with Gill's 1 hematoxylin, dried, cleared and cover-slipped.

## Discussion

Robust and reliable models are essential for efficient vaccine evaluation. For RVFV, there are currently a variety of effective small animal, NHP, sheep and goat models, although cattle have proven more difficult to reliably infect. In this study, successful infection of 3–6 month old Holstein calves with three different inoculation routes was demonstrated: intradermal (ID), intranasal (IN) and a combination of intradermal, intranasal and subcutaneous (SQ/ID/IN). All three routes reliably elicited viremia, with the combination and ID routes producing similar viral titers while the IN route generated much higher viral titers. The clinical scores for each group correlated strongly with the intensity of viremia, where higher clinical scores and rectal temperatures were seen in the IN group whereas mild to asymptomatic clinical scores were observed in the ID and combination groups.

Clinical biochemistry markers were evaluated to monitor organ function throughout infection which indicted mild increases in albumin (ALB), total protein (TP), alkaline phosphatase (ALP) and calcium (CA) in all groups and elevated blood urea nitrogen (BUN) levels in the intranasal group. Interestingly, a more prominent elevated level of ALP was detected in Wilson's cattle model in both SA01 and Ken06 infected groups, but not in the uninfected control group (57). The mild increases in our current model could potentially be due to bone growth as our cattle are still growing; however, in light of Wilson's data it is also possible that the increase could be due to the infection. More data is needed to evaluate this further. Other clinical chemistry values that were elevated in this study include ALB, TP, and CA, which might indicate mild dehydration; the same mild elevations were not found in Wilson's study (57). Another change that was specific to the intranasal group was an increase in BUN levels. One cause of elevated BUN levels could be dehydration; however since only the intranasal group was affected and only the intranasal group had infectious virus isolated from the kidney, this may again be RVFV specific. Future experiments could include a urinalysis to confirm this. During the necropsy, it was noted that calf 1,835 had significant areas of portal fibrosis and bile duct hyperplasia in the liver which likely caused impairment of liver function prior to arriving at our facility and explains the high GGT values in that specific animal. Liver necrosis was also detected in all animals; however, as liver enzyme levels such as AST were not considered clinically abnormal, the extent of liver damage was likely not extensive enough to compromise organ function.

Interestingly, infectious virus was found in the turbinates of all three groups, suggesting that the nasal swabs could contain virus. Yet, only the intranasal group had detectable viral RNA in the nasal swabs and none of them contained infectious virus. These results were consistent with previous reports in Nubian goats (55); however, one other study has reported infectious virus in nasal swabs (57). While assaying potential virus neutralizing components in swabs, we found both ID and IN infections induced IFN-β and IFN-γ secretion, but only the IN infection contained IFN-α in nasal swabs. Many cell types can secrete interferons, which then act on the same or nearby cells to induce an intracellular antiviral state. Therefore, the presence of interferons in the swabs could indicate that the nasal and oral mucosal environment may be able to prevent active replication of RVFV through activation of an antiviral state via interferon and copies of viral RNA detected may represent incomplete virus found in the cytoplasm of cells. Alternatively, other components that were not measured may also be present in the swabs that are able to neutralize infectious virus, such as antibodies.

The only tissue to be consistently infected in all three experimental groups was the turbinate. The IN infection was much more widespread than the other groups and had a higher number of infected tissues, which may have been due to the increased titers and duration of viremia in this group. Perhaps most strikingly, some brain tissues and the cerebral spinal fluid (CSF) in one animal produced infectious virus in the IN group but not in the other groups, at least as measured at 4 days post infection. Other studies have identified RVFV in brain tissues as well, although it is unusual to find data on different areas of the brain. For example, RVFV was isolated from the brains of 21 day old calves infected subcutaneously with RVFV (60), 4 month old calves infected subcutaneously with RVFV (57) or 4 month old goats infected subcutaneously with RVFV (55). In addition, clinical neurological manifestations could be readily seen in young 21 day old calves (60), but not in the 4 month old animals.

In comparison to Wilson's previous cattle model study, it was hypothesized that the success in infecting all animals was due to differences in virus dose, isolate, or cattle breed. Unfortunately there is no information about how the pathogenicity compares between the Kenya-128b isolate used in Wilson's study and the RVFV-UAP isolate used in the current study. The two isolates were shown to be phylogenetically distinct but still very similar in sequence (55). Any sequence or amino acid changes did not fall within critical sites that have been characterized such as the RNA polymerase active site or phosphorylation sites, although point mutations are not well-characterized in RVFV and their effects are unknown. It is also difficult to speculate whether the Holstein cattle breed was more susceptible to RVFV than the Angus breed as we did not directly compare the two with the same parameters. While more groups and more comparisons would have been scientifically interesting, we could not justify all of them due to the number of animals required. Since the focus was on developing an effective challenge model, parameters were chosen that would most likely produce a reliable challenge model with viremia.

Age is an important factor in RVFV cattle infections as both disease severity and the ability to mount an immune response to a vaccine are age dependent. For example, young ruminant neonates (1–2 months) are highly susceptible to RVFV with mortality rates of up to 100% and would demonstrate a severe RVFV infection. However, the goal was to create a RVFV infection for testing vaccines at an age with a mature immune system. It was previously demonstrated that 3–6 month-old sheep, goats and cattle all mount robust immune responses against RVFV infection (54–57). Perhaps because of these strong immune responses, the overall disease severity in our model was quite mild, especially considering the fact that RVFV can be lethal to adult ruminants during outbreaks. In this respect, it is also worth considering the fact that our animals were all of high health status, well-fed, in temperature-controlled housing, free of any obvious underlying disease, and free of many stressors.

## Conclusion

This study was conducted to determine an optimal RVFV infection in cattle for vaccine efficacy studies. Overall, it was demonstrated that RVFV infection could be achieved via three different routes of infection in vaccine-aged cattle using an endpoint at 4 days post infection. This day coincides with the peak of infection and is ideal to compare vaccinated to non-vaccinated animals. Interestingly, all three routes were effective at inducing viremia and producing liver lesions, which are two major hallmarks of RVFV infection. However, a major difference between the groups consisted of increased systemic spread of the virus to tissues in the intranasal group by 4 dpi, which was much less pronounced in the other groups. As the intranasal route is not thought to be a natural route of infection for livestock, the intradermal or subcutaneous models may mimic a natural infection more closely. However, the intranasal route generated the most severe clinical disease and most robust virus replication, making it an excellent challenge model to use to evaluate the ability of RVFV vaccines to decrease viremia in cattle.

## Data Availability Statement

All datasets generated for this study are included in the article/Supplementary Material.

## Ethics Statement

The animal study was reviewed and approved by CACC.

## Author Contributions

Conceptualization, data curation, writing—original draft preparation, visualization, and project administration: AK. Methodology: MP. Formal analysis: AK and CE-H. Investigation: AK, VS, and BC. Writing—review and editing and supervision: AK, BP, and SB. Funding acquisition: BP and SB.

## Conflict of Interest

The authors declare that the research was conducted in the absence of any commercial or financial relationships that could be construed as a potential conflict of interest.

## Supplementary Material

Supplementary Figure 1 Viral loads in serum and tissues.
