# Supplementary material for: Increased Susceptibility of Cattle to Intranasal RVFV Infection
Source: Front Vet Sci. 2020 Apr 29;7:137. doi: 10.3389/fvets.2020.00137 (PMC7200984; doi:10.3389/fvets.2020.00137)
Supplement: Supplementary file 1 [file Table_1.docx]

**Supplementary Table 1 – Clinical scores**

|  |  | **Intradermal group**  **Calf # 1904/1911/1912/1913** | | | | | **Intranasal group**  **Calf # 1833/1835/1836/1903** | | | | | **Combination group**  **Calf # 1801/1805/1818/1820** | | | | |  |  |  |  |  |  |  |  |  |
| --- | --- | --- | --- | --- | --- | --- | --- | --- | --- | --- | --- | --- | --- | --- | --- | --- | --- | --- | --- | --- | --- | --- | --- | --- | --- |
|  |  | **DPI**  **0** | **DPI**  **1** | **DPI**  **2** | **DPI**  **3** | **DPI**  **4** | **DPI**  **0** | **DPI**  **1** | **DPI**  **2** | **DPI**  **3** | **DPI**  **4** | **DPI**  **0** | **DPI**  **1** | **DPI**  **2** | **DPI**  **3** | **DPI**  **4** |  |  |  |  |  |  |  |  |  |
| ***Appearance*** | | | | | | | | | | | | | | | | |  |  |  |  |  |  |  |  |  |
| normal | 0 | 0/0/0/0 | 0/0/0/0 | 0/0/0/0 | 0/0/0/0 | 0/0/0/0 | 0/0/0/0 | 0/0/0/0 | .5/.5/.5/.5 | 2/2/.5/.5 | 2/2/2/2 | 0/0/0/0 | 0/0/0/0 | 0/0/0/0 | 0/0/0/0 | 0/0/0/0 |  |  |  |  |  |  |  |  |  |
| mild disease | 1 |  |  |  |  |  |  |  |  |  |  |  |  |  |  |  |  |  |  |  |  |  |  |  |  |
| disease | 2 |  |  |  |  |  |  |  |  |  |  |  |  |  |  |  |  |  |  |  |  |  |  |  |  |
| Fever (>40C) | 0/1 | 0/0/0/0 | 1/0/1/0 | 0/1/1/1 | 0/1/0/1 | 0/1/0/1 | 0/0/0/0 | 0/0/0/0 | 0/0/0/0 | 1/1/0/1 | 1/1/1/1 | 0/0/0/0 | 1/0/1/1 | 0/1/0/0 | 1/0/1/0 | 0/0/0/1 |  |  |  |  |  |  |  |  |  |
| ***Disposition*** | | | | | | | | | | | | | | | | |  |  |  |  |  |  |  |  |  |
| BAR | 0 | 0/0/0/0 | 0/0/0/0 | 0/0/0/0 | 0/1/0/1 | 0/0/0/0 | 0/0/0/0 | .5/.5/.5/.5 | .5/.5/.5/.5 | 1/1/1/1 | 1.5/1.5/1.5/1.5 | 0/0/0/0 | 1/1/1/1 | 2/2/2/2 | 2/2/2/2 | 1/1/1/1 |  |  |  |  |  |  |  |  |  |
| QAR | 1 |  |  |  |  |  |  |  |  |  |  |  |  |  |  |  |  |  |  |  |  |  |  |  |  |
| depressed | 2 |  |  |  |  |  |  |  |  |  |  |  |  |  |  |  |  |  |  |  |  |  |  |  |  |
| ***Eating*** | | | | | | | | | | | | | | | | |  |  |  |  |  |  |  |  |  |
| normal | 0 | 0/0/0/0 | 0/0/0/0 | 0/0/0/0 | 0/1/0/1 | 0/1/0/1 | 0/0/0/0 | 0/0/0/0 | 0/0/0/0 | 1/1/1/1 | 1/1/1/1 | 0/0/0/0 | 0/0/0/0 | 0/0/0/0 | 0/0/0/0 | 0/0/0/0 |  |  |  |  |  |  |  |  |  |
| some eating | 1 |  |  |  |  |  |  |  |  |  |  |  |  |  |  |  |  |  |  |  |  |  |  |  |  |
| little eating | 2 |  |  |  |  |  |  |  |  |  |  |  |  |  |  |  |  |  |  |  |  |  |  |  |  |
| ***Drinking*** | | | | | | | | | | | | | | | | |  |  |  |  |  |  |  |  |  |
| normal | 0 | 0/0/0/0 | 0/0/0/0 | 0/0/0/0 | 0/0/0/0 | 0/0/0/0 | 0/0/0/0 | 0/0/0/0 | 0/0/0/0 | 0/0/0/0 | 0/0/0/0 | 0/0/0/0 | 0/0/0/0 | 0/0/0/0 | 0/0/0/0 | 0/0/0/0 |  |  |  |  |  |  |  |  |  |
| not drinking | 2 |  |  |  |  |  |  |  |  |  |  |  |  |  |  |  |  |  |  |  |  |  |  |  |  |
| ***Feces*** | | | | | | | | | | | | | | | | |  |  |  |  |  |  |  |  |  |
| stool normal | 0 | 0/0/0/0 | 0/0/0/0 | 0/1/0/0 | 0/0/0/0 | 0/0/0/0 | 0/0/0/0 | 1.5/1.5/1.5/1.5 | 1.5/1.5/1.5/1.5 | 1.5/1.5/1.5/1.5 | 1.5/1.5/1.5/1.5 | 0/0/0/0 | 0/0/0/0 | 0/0/0/0 | 0/0/0/0 | 0/0/0/0 |  |  |  |  |  |  |  |  |  |
| <50% soft | 1 |  |  |  |  |  |  |  |  |  |  |  |  |  |  |  |  |  |  |  |  |  |  |  |  |
| >50% soft | 1.5 |  |  |  |  |  |  |  |  |  |  |  |  |  |  |  |  |  |  |  |  |  |  |  |  |
| diarrhea | 2 |  |  |  |  |  |  |  |  |  |  |  |  |  |  |  |  |  |  |  |  |  |  |  |  |
| **MAX SCORE** | **11** | 0/0/0/0 | 1/0/1/0 | 0/2/1/1 | 0/3/0/3 | 0/2/0/2 | 0/0/0/0 | 2/2/2/2 | 2.5/2.5/2.5/2.5 | 6/6/3.5/4.5 | 7/7/7/7 | 0/0/0/0 | 2/1/2/2 | 2/3/2/2 | 3/2/3/2 | 1/1/1/2 |  |  |  |  |  |  |  |  |  |

**Supplementary Table 2 – Clinical chemistry**

| **Intradermal** | | | | | | | | | | | | | | | | | | | | | | | | | | | |  |
| --- | --- | --- | --- | --- | --- | --- | --- | --- | --- | --- | --- | --- | --- | --- | --- | --- | --- | --- | --- | --- | --- | --- | --- | --- | --- | --- | --- | --- |
|  | **Calf #1904 (8 mths)** | | | | | **Calf #1911 (7 mths)** | | | | | **Calf #1912 (6 mths)** | | | | | | | **Calf #1913 (5 mths)** | | | | | | | | | |  |
|  | **DPI 0** | **DPI 1** | **DPI 2** | **DPI 3** | DPI 4 | DPI 0 | DPI 1 | DPI 2 | DPI 3 | DPI 4 | DPI 0 | DPI 1 | DPI 2 | | DPI 3 | DPI 4 | | DPI 0 | | DPI 1 | | DPI 2 | | DPI 3 | | DPI 4 | |  |
| **ALB** | 3.4 | 3.5 | 4.9 | 4.5 | 3.5 | 3.6 | 5.5 | 5.6 | 4 | 5 | 4.1 | 4.3 | 4.8 | | 3.5 | 3.5 | | 3.9 | | 5.4 | | 5.4 | | 3.8 | | 3.6 | |  |
| **ALP** | 120 | 169 | 241 | 182 | 139 | 179 | 202 | 214 | 130 | 163 | 154 | 220 | 258 | | 169 | 149 | | 137 | | 214 | | 234 | | 160 | | 131 | |  |
| **AST** | 59 | 62 | 92 | 75 | 54 | 75 | 125 | 176 | 85 | 107 | 66 | 123 | 134 | | 74 | 61 | | 81 | | 124 | | 114 | | 69 | | 49 | |  |
| **CA** | 10 | 10.3 | 15.1 | 13.5 | 10.7 | 11.1 | 15.7 | 15.8 | 11.6 | 14.5 | 9.9 | 13.2 | 15 | | 11.2 | 11 | | 12 | | 15.7 | | 15.4 | | 11.2 | | 10.6 | |  |
| **GGT** | 15 | 17 | 29 | 18 | 17 | 17 | 29 | 31 | 20 | 27 | 16 | 26 | 30 | | 21 | 21 | | 18 | | 19 | | 22 | | 15 | | 15 | |  |
| **TP** | 7 | 6.8 | 10.5 | 9.4 | 7 | 8.2 | 10.5 | 10.9 | 7.1 | 9.6 | 7.5 | 8.8 | 12.6 | | 7.1 | 6.9 | | 7.7 | | 10.7 | | 10.5 | | 6.9 | | 6.5 | |  |
| **GLOB** | 4 | 3.3 | 5.6 | 4.9 | 3.5 | 3.5 | 5 | 5.4 | 3.1 | 4.5 | 3.2 | 4.4 | 5.8 | | 3.6 | 3.5 | | 3.3 | | 5.3 | | 5 | | 3.1 | | 2.9 | |  |
| **BUN** | 12 | 12 | 22 | 13 | 11 | 18 | 14 | 13 | 9 | 11 | 16 | 13 | 17 | | 12 | 8 | | 11 | | 11 | | 9 | | 9 | | 7 | |  |
| **CK** | 235 | 253 | 353 | 298 | 286 | 235 | 330 | 344 | 231 | 258 | 456 | 503 | 825 | | 286 | 254 | | 354 | | 663 | | 408 | | 637 | | 212 | |  |
| **PHOS** | 6.7 | 8.1 | 15 | 9.9 | 9.4 | 7.2 | 9.7 | 7.2 | 6.5 | 7.7 | 6 | 9.1 | 10.6 | | 6.8 | 6.7 | | 8.1 | | 9.1 | | 8.8 | | 6.7 | | 6.6 | |  |
| **MG** | 2.2 | 2 | 3.5 | 2.8 | 2.2 | 2.8 | 3.1 | 3.3 | 2.1 | 2.7 | 2.4 | 2.5 | 3.1 | | 2.2 | 2.3 | | 2.1 | | 3.3 | | 3.1 | | 2.1 | | 1.9 | |  |
| **Intranasal** | | | | | | | | | | | | | | | | | | | | | | | | | | | |  |
|  | **Calf #1833 (5 mths)** | | | | | **Calf #1835 (4.5 mths)** | | | | | **Calf #1836 (4.5 mths)** | | | | | | **Calf #1903 (3 mths)** | | | | | | | | | | |  |
|  | DPI 0 | DPI 1 | DPI 2 | DPI 3 | DPI 4 | DPI 0 | DPI 1 | DPI 2 | DPI 3 | DPI 4 | DPI 0 | DPI 1 | | DPI 2 | DPI 3 | DPI 4 | DPI 0 | | | DPI 1 | | DPI 2 | | DPI 3 | | DPI 4 | |  |
| **ALB** | 3.4 | 5.4 | 3.8 | 4.0 | 4.3 | 3.7 | 4.4 | 3.7 | 3.5 | 3.4 | 3.7 | 4.8 | | 4.5 | 4.0 | 5.1 | 3.9 | | | 4.6 | | 3.9 | | 3.6 | | 4.7 | |  |
| **ALP** | 115 | 184 | 109 | 117 | 136 | 170 | 196 | 162 | 152 | 204 | 154 | 214 | | 171 | 172 | 249 | 105 | | | 130 | | 110 | | 108 | | 143 | |  |
| **AST** | 57 | 124 | 72 | 77 | 130 | 72 | 99 | 84 | 110 | 178 | 59 | 87 | | 74 | 261 | 249 | 66 | | | 87 | | 84 | | 121 | | 200 | |  |
| **CA** | 10.5 | >16 | 11.0 | 10.7 | 11.7 | 11.8 | 13.7 | 12.1 | 11.3 | 11.0 | 11.7 | 14.7 | | 13.5 | 11.6 | 14.6 | 11.1 | | | 13.5 | | 11.4 | | 10.4 | | 12.9 | |  |
| **GGT** | 12 | 20 | 12 | 14 | 19 | 309 | 331 | 257 | 214 | 231 | 17 | 27 | | 19 | 25 | 38 | 18 | | | 22 | | 15 | | 20 | | 31 | |  |
| **TP** | 6.4 | 12.1 | 7.0 | 7.5 | 8.7 | 7.7 | 9.5 | 7.8 | 7.1 | 7.0 | 7.4 | 10.3 | | 8.4 | 7.6 | 10.2 | 7.8 | | | 9.7 | | 7.8 | | 7.2 | | 10.1 | |  |
| **GLOB** | 3.0 | 6.7 | 3.1 | 3.5 | 4.3 | 4.0 | 5.2 | 4.1 | 3.5 | 3.6 | 3.7 | 5.5 | | 4.0 | 3.6 | 5.2 | 3.9 | | | 5.1 | | 3.9 | | 3.6 | | 5.3 | |  |
| **BUN** | 17 | 29 | 17 | 20 | 24 | 18 | 19 | 15 | 14 | 20 | 16 | 21 | | 18 | 24 | 30 | 15 | | | 22 | | 17 | | 17 | | 25 | |  |
| **CK** | 244 | 534 | 339 | 356 | 418 | 317 | 476 | 571 | 369 | 327 | 274 | 380 | | 384 | 256 | 381 | 291 | | | 386 | | 924 | | 268 | | 447 | |  |
| **PHOS** | 6.9 | 10.5 | 8.5 | 6.4 | 6.0 | 6.5 | 7.3 | 5.7 | 5.6 | 6.9 | 6.0 | 7.8 | | 6.8 | 5.4 | 10.4 | 7.5 | | | 7.3 | | 8.2 | | 5.4 | | 10 | |  |
| **MG** | 2.1 | 4.2 | 2.2 | 2.0 | 2.3 | 2.3 | 2.0 | 2.3 | 1.8 | 2.1 | 2.3 | 3.3 | | 2.5 | 2.2 | 2.9 | 2.4 | | | 3.0 | | 2.3 | | 1.9 | | 2.7 | |  |
| **SQ-ID-IN combination** | | | | | | | | | | | | | | | | | | | | | | | | | | | | |
|  | **Calf #1801 (8 mths)** | | | | | **Calf #1805 (6 mths)** | | | | | **Calf #1818 (4 mths)** | | | | | | | | **Calf #1820 (4 mths)** | | | | | | | | | |
|  | **DPI 0** | **DPI 1** | **DPI 2** | **DPI 3** | **DPI 4** | **DPI 0** | **DPI 1** | **DPI 2** | **DPI 3** | **DPI 4** | **DPI 0** | **DPI 1** | | **DPI 2** | **DPI 3** | **DPI 4** | | | **DPI 0** | | **DPI 1** | | **DPI 2** | | **DPI 3** | | **DPI 4** | |
| **ALB** | 5.5 | 3.6 | 3.4 | 4.2 | 4.8 | 3.8 | 4.3 | 3.4 | 4.2 | 4.5 | 5.6 | 3.9 | | 4.1 | 4.5 | 5.5 | | | 6.4 | | 5.7 | | 4.4 | | 4.2 | | 3.6 | |
| **ALP** | 205 | 114 | 105 | 126 | 143 | 122 | 133 | 100 | 115 | 108 | 284 | 178 | | 179 | 178 | 204 | | | 285 | | 205 | | 159 | | 138 | | 104 | |
| **AST** | 122 | 70 | 64 | 79 | 99 | 75 | 90 | 68 | 81 | 82 | 131 | 78 | | 79 | 74 | 92 | | | 114 | | 106 | | 74 | | 65 | | 88 | |
| **CA** | >16 | 10.3 | 10 | 12.5 | 14.2 | 12.2 | 13.4 | 10.4 | 12.8 | 13.8 | >16 | 1.5 | | 12.1 | 13.5 | 15.6 | | | >16 | | >16 | | 12.7 | | 12.9 | | 10.8 | |
| **GGT** | 36 | 18 | 18 | 20 | 26 | 25 | 27 | 21 | 26 | 30 | 24 | 12 | | 15 | 19 | 20 | | | 25 | | 22 | | 16 | | 16 | | 14 | |
| **TP** | 3.4 | 7.2 | 7 | 8.9 | 10.9 | 7.9 | 9.4 | 7.1 | 9.1 | 10 | 11.5 | 7.4 | | 7.8 | 9 | 11.4 | | | 14 | | 11.5 | | 8.2 | | 7.7 | | 6.4 | |
| **GLOB** | 7.9 | 3.6 | 3.7 | 4.7 | 6.1 | 4.1 | 5.1 | 3.7 | 4.9 | 5.5 | 5.9 | 3.4 | | 3.7 | 4.6 | 5.9 | | | 7.5 | | 5.8 | | 3.8 | | 3.5 | | 2.8 | |
| **BUN** | 20 | 12 | 13 | 15 | 19 | 13 | 15 | 13 | 17 | 17 | 25 | 17 | | 20 | 21 | 19 | | | 28 | | 22 | | 20 | | 16 | | 11 | |
| **CK** | 620 | 376 | 409 | 490 | 567 | 395 | 419 | 328 | 399 | 413 | 342 | 254 | | 281 | 302 | 346 | | | 336 | | 271 | | 207 | | 207 | | 137 | |
| **PHOS** | 13.7 | 7.3 | 7.9 | 10 | 12 | 8.7 | 10 | 7.5 | 9.2 | 12.1 | 13.5 | 8.2 | | 9.1 | 9.9 | 12.8 | | | 10.9 | | 9.7 | | 8 | | 7.5 | | 6 | |
| **MG** | 4 | 2.1 | 2.1 | 2.7 | 3.5 | 2.4 | 2.7 | 2.2 | 2.5 | 2.8 | 4 | 2.2 | | 2.6 | 2.8 | 3.9 | | | 4 | | 2.9 | | 2.3 | | 2.1 | | 1.8 | |
