# Supplementary figures and images for: Increased Susceptibility of Cattle to Intranasal RVFV Infection
Source: Front Vet Sci. 2020 Apr 29;7:137. doi: 10.3389/fvets.2020.00137 (PMC7200984; doi:10.3389/fvets.2020.00137)

## Slide 1
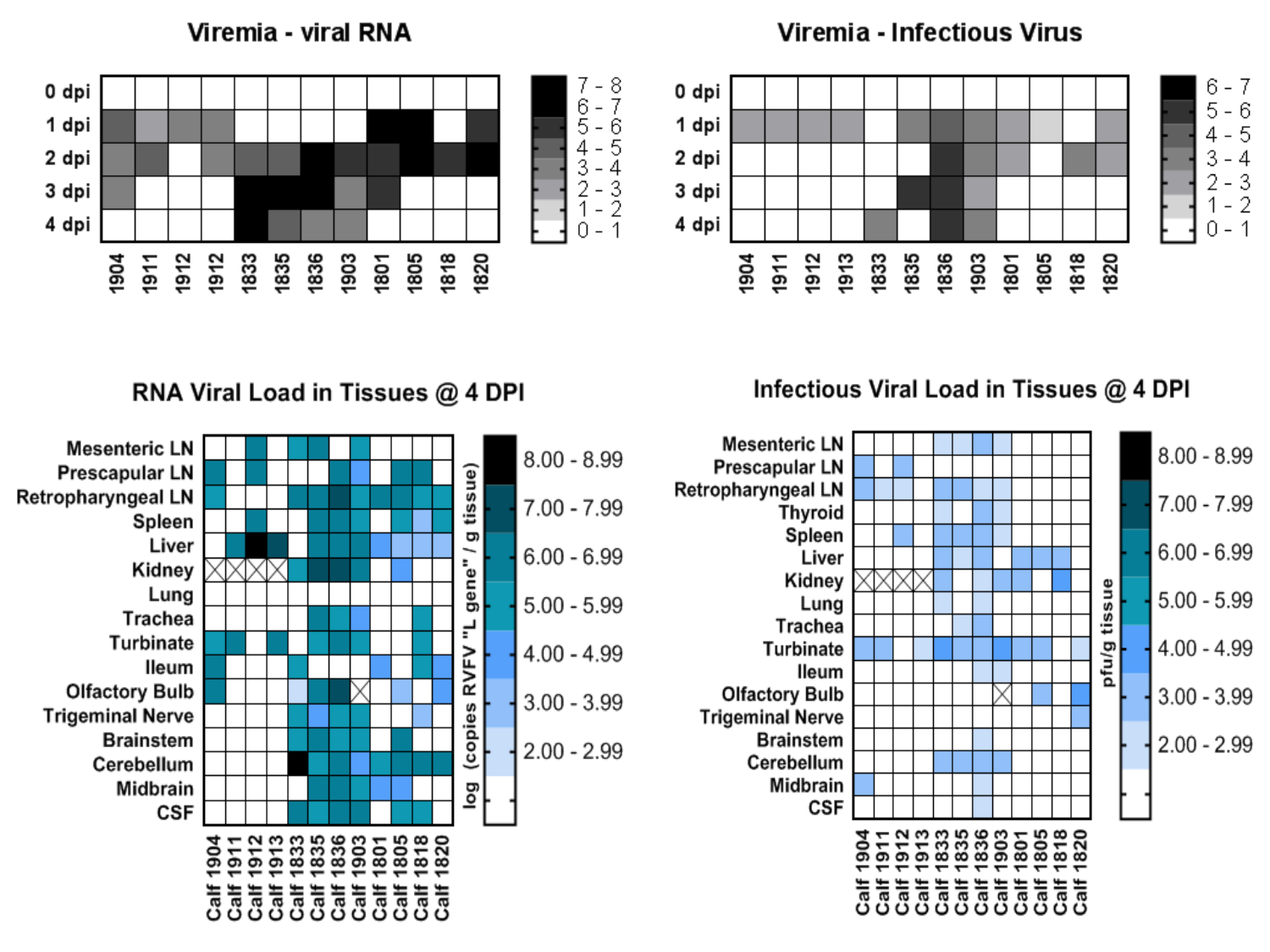

Supplement: Supplementary Figure 1 — Viral loads in serum and tissues. [file Presentation_1.PPTX]
